# Genetic analysis of African swine fever viruses based on E183L (p54) gene, circulating in South Sumatra and Lampung province, Indonesia

**DOI:** 10.14202/vetworld.2023.1985-1990

**Published:** 2023-09-23

**Authors:** Fransiska Panasea Anggy, Widagdo Sri Nugroho, Sri Handayani Irianingsih, Saswiyanti Enny, Eko Agus Srihanto

**Affiliations:** 1 Disease Investigation Center Lampung, Lampung, Indonesia; 2 Department of Veterinary Public Health, Faculty of Veterinary Medicine, Universitas Gadjah Mada, Yogyakarta, Indonesia; 3 Disease Investigation Center Wates, Yogyakarta, Indonesia

**Keywords:** African swine fever virus genotype, Lampung, phylogenetic tree, South Sumatra

## Abstract

**Background and Aim:**

African swine fever (ASF) is a disease that emerged in Indonesia in 2019 in the North Sumatra province and spread rapidly to other areas, such as South Sumatra and Lampung, in 2020. This study aimed to identify the phylogenetics of the ASF virus (ASFV) in the provinces of South Sumatra and Lampung.

**Materials and Methods:**

Nine ASFV isolates collected from the Disease Investigation Center in Lampung were used in this study. The isolates were from ASF cases in South Sumatra and Lampung in 2020–2022. The isolates were sequenced and compared with other ASFV isolates to establish the virus genotype. Sequencing was performed using the complete E183L gene target encoding the p54 protein.

**Results:**

This study showed that ASFV from South Sumatera and Lampung Province belongs to genotype II.

**Conclusion:**

Based on the analysis of the E183L gene, all nine ASFV isolates that originated from South Sumatra and Lampung were identical to other genotype II ASFV isolates from Georgia, China, Vietnam, and Timor Leste.

## Introduction

African swine fever virus (ASFV) is the cause of African swine fever (ASF), which causes hemorrhagic fever in domestic pigs and wild boars. The virus belongs to the Asfarviridae family and genus *Asfivirus* [1]. African swine fever virus is an enveloped deoxyribonucleic acid (DNA) virus with 70–100 nm nucleoproteins surrounded by a lipid layer and an isohedral capsid measuring 170–190 nm in diameter. The viral genome is a sizable single-stranded DNA in the range of 170 kb–190 kb, which includes approximately 150 open reading frames and encodes various enzymes that play a role in structural protein, DNA replication, gene transcription, and protein modification. This virus has approximately 50 proteins [2]. This intracellular virus has four structural layers: nucleoid, protein shell core, inner lipid envelope, and protein capsid [3]. The clinical signs of the disease vary from acute to chronic [4]. The reported mortality rate is 100% [5]. The high mortality rate, socioeconomic impact, and unavailability of an effective vaccine make ASF one of the most dangerous animal diseases [6]. The transmission route of the virus is divided into sylvatic, domestic, and wild boar cycles. The sylvatic cycle exists exclusively in Africa and involves *Ornithodoros moubata* as a biological vector. The domestic and wild boar cycles have been reported outside Africa without the involvement of *O. moubata* [7]. The domestic cycle involves domestic pig and their by-products in the transmission route, whereas the wild boar cycle includes the wild boars in the absence of *O. moubata* ticks [8]. The unavailability of an effective vaccine against the disease results in disease control by animal quarantine and culling [9]. Virus detection on the ASF-suspected samples was performed using the real-time polymerase chain reaction (PCR) method as the gold standard set by World Organization for Animal Health (WOAH). The target gene for this assay is the VP72 gene in the conserved region to ensure that the assay can detect various ASFV isolates [10].

Indonesia reported the first ASF outbreak in early September 2019 in North Sumatra. The disease continuously spread to South Sumatra in March 2020 and Lampung in July 2020. No study has been published regarding the genetic variation analysis of ASFV, which was responsible for the outbreak in South Sumatra and Lampung. The genetic variation in ASFV can be analyzed based on specific genetic targets, such as the partial B646L gene, which encodes p72 capsid protein, and the E183L gene, which encodes envelope protein 54 (p54). Partial p72 gene analysis classified ASF viruses into 24 genotypes, but it did not provide sufficient genotyping resolution nor the capability to distinguish between biologically phenotypically different viruses [11]. The E183L gene that encodes the p54 protein was analyzed entirely with a 767 bp length. The p54 protein has been successfully used to classify isolates from West and East Africa, Europe, and America into four different genotypes I subclusters, and the East Africa ASFV isolate genotypes V, X, and XX were grouped into Va, Vb, Xa, Xbm, XXa, and XXb subclusters. This suggests that a high level of resolution of viral differentiation can be obtained using p54 gene sequencing [12].

This study aimed to analyze the genetics of the ASFV that circulated in South Sumatra and Lampung in 2019–2022 based on the E183L gene and to determine the genetic relationship of this virus with ASFVs from other countries. The previous study on the genetic variation of ASFV in Indonesia was based on the B464L gene observed in North Sumatra and West Java [13]. No research has been conducted on genetic variation of the ASFV in the provinces of South Sumatra and Lampung. This study also used samples from native wild pigs, which had not been previously conducted in Indonesia.

## Materials and Methods

### Ethical approval

This study was approved by Disease Investigation Centre, Lampung, Indonesia (No. 00156/SPT/KU.300/025/F5C/06/2020).

### Study period and location

This study was conducted from June to October 2022 at the Disease Investigation Center (DIC) Lampung. South Sumatra and Lampung were involved in the DIC Lampung working area.

### Sample collection

A total of 15 samples were collected from five districts: South Lampung, East Lampung, and Central Lampung (domestic pigs); Muara Enim, and North Musi Rawas (wild boars). The samples were obtained from outbreaks of ASF from 2020–2022 based on the DIC Lampung report. Domestic pigs presented several clinical symptoms, such as anorexia, hemorrhage in extremities and chest muscles, and sudden death. Organ samples, spleen, and liver were removed from the dead pigs, and 3 mL blood samples were collected from the vena cava of live pigs and put with ethylene diamine tetraacetic acid in the tube. All samples were stored at −80°C until laboratory testing. The samples from wild boars, which were obtained using bone marrow from wild boar carcasses, were found by natural resource conservation officers. The sample sent was in the form of a long bone which probably came from the extremity of a wild boar.

### DNA extraction and amplification

The DNA was extracted from the samples using QIAamp DNA Mini Kit (Qiagen, Germany), and the presence of ASFV was confirmed using a real-time PCR method targeting VP72 gene based on the following primers: forward primer 5’-CTGCTCATGGTATCAATCTTATCGA-3’, reverse primer 5’-GATACCACAAGATCRGCCGT-3’, and probe 5’-FAM-CCACGGGAGGAATACCAAC CCAGTG-TAMRA-3’ [10]. This step ensured the sample quality based on the cycle threshold (CT) value. The master mix reagent used for each reaction consisted of 4.6 μL nuclease-free water, 10 μL SensiFAST™ Probe Lo-ROX Kit (Bioline, UK), 0.8 μL primer forward (20 pmol/μL), 0.8 μL primer reverse (20 pmol/μL), and 0.8 μL probe (5 pmol/μL). The volume of DNA used as a template was 3 μL. The amplification process was carried out on an ABI 7500 real-time PCR system (Applied Biosystem, USA) using the following program: 60°C for 30 s; 95°C for 5 min; 40 cycles of 95°C for 10 s; and 60°C for 50 s. The fluorescent signal was determined at the end of each cycle. The expected positive result was a CT value under 38.0 with a cutoff value of 0.05.

### E183L gene sequencing

The DNA product from samples with positive results in the real-time PCR test was subjected to amplification of the E183L gene using the following primers: forward primer 5’-GGCACAAGTTCGGACATGT-3’ and reverse primer 5’-GTACTGTAACGCAGCACAG-3’. The master mix reagent consisted of 12 μL nuclease-free water, 20 μL MyTaq™ Red Mix (Bioline), 1.5 μL primer forward, and 1.5 μL primer reverse. The primer concentration was used on 20 pmol/-μL. A total of 5 μL DNA volume was used as a template. The thermocycler machine was prepared using the following program: predenaturation (94°C for 7 min) for one cycle; (denaturation [temperature 94°C for 15 s]; annealing [temperature 56°C for 45 s]; elongation [72°C for 60 s]) for 35 cycles; and post elongation (72°C for 7 min) for one cycle. The PCR products were then electrophoresed with a size of 676 bp. Samples that showed appropriate DNA bands were used for E183L gene sequencing. If two samples originated from a similar farm, sequencing was performed on only one of them. Sanger sequencing was used as the sequencing method. The ABI PRISMA 3730xl (Applied Biosystem) was used to analyze samples. The sequencing kit was a BigDye Terminator v3.1 (Applied Biosystem).

### Phylogenetic analysis

UGENE v.3.7.0 (Unipro, Russia) and MEGA XI software (MEGA Software) were used to analyze the DNA sequences. All ASFV reference sequences were downloaded from GenBank at the National Center for Biotechnology Information. Nucleotide sequence correction was performed using UGENE v.3.7.0 software, and phylogenetic analysis was performed using MEGA XI software. The phylogenetic tree was made by the maximum likelihood method.

## Results

### Specimen

Positive field samples for the ASFV, namely, 15 samples from three districts in Lampung and two districts in South Sumatra directly adjacent to Lampung, were obtained during 2019–2022. Regencies in Lampung that have positively confirmed ASF are South Lampung Regency, East Lampung Regency, and Central Lampung Regency. Samples for these regencies were collected from domestic pigs. The regencies of South Sumatera Province whose samples were analyzed were those of North Musi Rawas and Muara Enim regencies. Samples from the two regencies were collected from wild pigs.

### Real-time PCR

Real-time PCR tests of the samples showed positive results for the ASFV with varying CT values (Table-1). The cutoff value was 0.05, with a CT value below 37.0 interpreted as positive.

**Table-1:** Result of real-time PCR test of African swine fever virus.

Sample code	Sample origin	CT value	Interpretation
001	South Lampung	15.82	Positive
002	South Lampung	26.96	Positive
003	South Lampung	20.44	Positive
004	South Lampung	26.31	Positive
005	South Lampung	15.76	Positive
006	South Lampung	16.84	Positive
007	South Lampung	18,78	Positive
008	South Lampung	18.82	Positive
009	South Lampung	16.9	Positive
010	South Lampung	15.12	Positive
011	East Lampung	17.82	Positive
012	East Lampung	16.29	Positive
013	North Musi Rawas	21.34	Positive
014	Muara Enim	21.11	Positive
015	West Lampung	14,4	Positive

PCR=Polymerase chain reaction, CT=Cycle threshold

### Genome analysis

**Figure-1:**
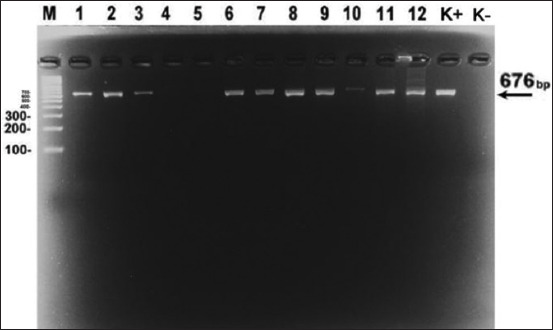
Electrophoretic visualization results of the E183L gene. Column 1 is a 100 bp marker, columns 2–13 are field samples, column 14 is a positive control, and column 15 is a negative control. The DNA band obtained was 676 bp in size.

In samples that showed positive results, the E183L gene (p54) was detected by conventional PCR method except for samples 007 and 008 because they were obtained from the same farm. The E183L detection had a DNA band with 676 bp in size (Figure-1). The sequenced samples were 001, 002, 005, 009, 012, 013, 014, and 015. The selection of the sequenced samples was based on the time and location of different outbreaks, and electrophoresis finding revealed a distinct DNA band.

**Figure-2:**
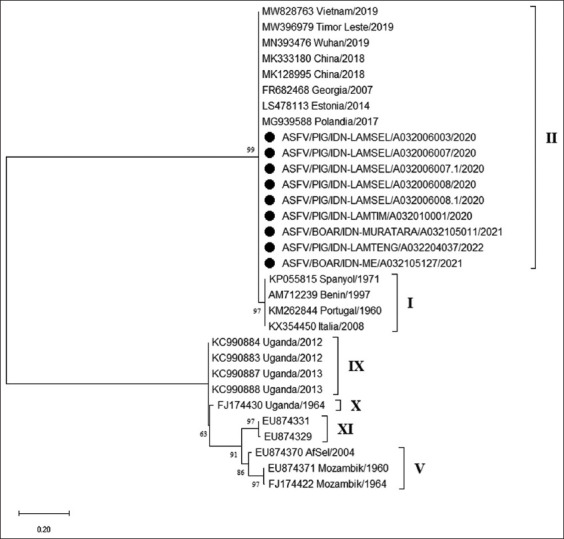
The phylogenetic tree of the African swine fever virus is based on the E183L gene (p54) with 676 bp in size. Phylogenetic analysis was performed using the maximum likelihood method, bootstrap value 1000, and K2+G model. The • sign indicates samples from this study.

The genetic relationship of the ASFV obtained against several ASFVs from other genotypes was analyzed by constructing a phylogenetic tree based on the p54 gene. The phylogenetic tree showed that all samples from Lampung and South Sumatera belong to genotype II (Figure-2). The genetic distances between all field sequences and between field sequences and other country sequences are shown in Tables-2 and 3, respectively.

**Table-2:** Genetic distance between all studied field sequences. The analysis using MEGA XI software.

Sample identity	1	2	3	4	5	6	7	8
1. ASFV/PIG/IDN-LAMSEL/A032006003/2020								
2. MW828763 Vietnam/2019	0.000							
3. MK128995 China/2018	0.000	0.000						
4. FR682468 Georgia/2007	0.000	0.000	0.000					
5. KX354450 Italia/2008	0.025	0.025	0.025	0.025				
6. EU874370 AfSel/2004	4.836	4.836	4.836	4.836	5.186			
7. KC990883 Uganda/2012	3.975	3.975	3.975	3.975	4.560	0.179		
8. FJ174430 Uganda/1964	4.665	4.665	4.665	4.665	5.477	0.185	0.020	
9. EU874331	4.108	4.108	4.108	4.108	4.739	0.094	0.194	0.200

**Table-3:** Genetic distance between the studied field sequence and somesequences from other different countries. The analysis using MEGA XI software.

Sample identity	1	2	3	4	5	6	7	8
10. ASFV/PIG/IDN-LAMTENG/A032204037/2022								
11. ASFV/BOAR/IDN-ME/A032105127/2021	0.000							
12. ASFV/BOAR/IDN-MURATARA/A032105011/2021	0.000	0.000						
13. ASFVPIG/IDN-LAMTIM/A032010001/2020	0.000	0.000	0.000					
14. ASFV/PIG/IDN-LAMSEL/A032006008.1/2020	0.000	0.000	0.000	0.000				
15. ASFV/PIG/IDN-LAMSEL/A032006008/2020	0.000	0.000	0.000	0.000	0.000			
16. ASFV/PIG/IDN-LAMSEL/A032006007.1/2020	0.000	0.000	0.000	0.000	0.000	0.000		
17. ASFV/PIG/IDN-LAMSEL/A032006007/2020	0.000	0.000	0.000	0.000	0.000	0.000	0.000	
18. ASFV/PIG/IDN-LAMSEL/A032006003/2020	0.000	0.000	0.000	0.000	0.000	0.000	0.000	0.000

## Discussion

African Swine Fever was first reported in Indonesia in 2019 in North Sumatera, and it spread rapidly to South Sumatera and Lampung in June 2020. A previous report by Dharmayanti *et al*. [13] mentioned that the ASFV belonging to genotype II that caused the outbreak in North Sumatra was the. Characterization of ASFV isolates helps increase the knowledge of the epidemiological pattern of the disease, which can help in planning effective prevention and disease control strategies and map different virus strains based on geographical location. This study verified that the isolates obtained from Lampung and South Sumatera are similar to those isolated in a previous report [13]. Nine E183L gene sequences were obtained in this study.

The p54 protein encoded by the E183L gene is a type II structural membrane protein located in the inner envelope of virions. This gene variation is not considered a virulence factor, but it helps in the classification of the virus into different genotypes. The p54 protein, together with the p30 protein, binds the virion to the target cell. After viral internalization, the p54 protein interacts with the host protein (dynein), which results in the transport of virions to the perinuclear area of the cell [14]. The corresponding dynein binding domain in the p54 protein also participated in capcase-3 activation and induction of apoptosis [14].

Phylogenetic analysis based on the E183L gene showed that all the isolates observed in this study belonged to the genotype II group. The genetic distance between all the field isolates showed a value of 0.000. This finding means that no difference existed between the ASFV sequences from domestic pigs and wild boars against the target gene. However, changes can only be determined using a complete genome analysis The route of infection of ASF in wild pigs can occur through several mechanisms, such as direct horizontal transmission, contact with contaminated environments, and involvement in human activities such as hunting [15]. This virus has a high case fatality rate in all infected pigs, domestic pigs, and wild boars [16].

African Swine Fever was first discovered in Kenya in 1921 [17]. This condition spread from Africa in 1957 to Europe and Portugal in 1960. The second transcontinental outbreak occurred in 2007 in Georgia, and it was caused by the highly virulent ASFV of genotype II [18]. The ASF then spread to Caucasian regional countries and the Russian Federation [19]. The disease reached Lithuania in 2014, followed by cases in several European Union countries [20]. African swine fever was first reported in China in 2018 [21, 22] and has spread to other Asian countries, including Vietnam (February 2019), Cambodia (March 2019), North Korea (May 2019), Laos (June 2019), Philippines (July 2019), Myanmar (August 2019), and South Korea and Timor Leste (September 2019) [23].

Genotype II ASFV has been the cause of widespread ASF disease outbreaks in Eastern Europe since 2007 and has spread to several countries, such as China, Vietnam, and Timor Leste. The genetic distance between the field isolates in this study and isolates from China, Vietnam, and Georgia was 0.000. This proved that the ASFV circulating in North Sumatra, South Sumatra, and Lampung Province is related to ASFV from China. However, the mechanism of its spread, whether through the illegal transportation of pigs and their products, is unknown. Thus, further investigation is required.

## Conclusion

Analysis of the E183L gene revealed that all nine ASFV isolates originating from South Sumatra and Lampung were identical to other genotype II ASFV isolates from Georgia, China, Vietnam, and Timor Leste. No difference was observed in the nucleotide sequences of ASFV in the target gene E183L between domestic pig and wild-boar virus isolates. Further research is required to determine the mechanism of entry of ASFV in Indonesia and the transmission route to wild boars.

## Authors’ Contributions

SE: Sample and data collection. FPA and EAS: Laboratory work, data analysis, and drafted the manuscript. WSN and SHI: Study design and drafted the manuscript. All authors have read, reviewed, and approved the final manuscript.
